# Cross-taxon congruence and environmental conditions

**DOI:** 10.1186/1472-6785-10-18

**Published:** 2010-07-16

**Authors:** Carolina Toranza, Matías Arim

**Affiliations:** 1 Instituto de Ecología y Ciencias Ambientales, Facultad de Ciencias, Universidad de la República, Uruguay, Iguá 4225 Piso 8 Sur, Montevideo, Uruguay; 2 Departamento de Ecología y Evolución, Facultad de Ciencias, Universidad de la República, Uruguay, Iguá 4225 Piso 9 Sur, Montevideo, Uruguay; 3 Center for Advanced Studies in Ecology and Biodiversity, Pontificia Universidad Católica de Chile, PO Box 114-D, Santiago, Chile

## Abstract

**Background:**

Diversity patterns of different taxa typically covary in space, a phenomenon called cross-taxon congruence. This pattern has been explained by the effect of one taxon diversity on taxon diversity, shared biogeographic histories of different taxa, and/or common responses to environmental conditions. A meta-analysis of the association between environment and diversity patterns found that in 83 out of 85 studies, more than 60% of the spatial variability in species richness was related to variables representing energy, water or their interaction. The role of the environment determining taxa diversity patterns leads us to hypothesize that this would explain the observed cross-taxon congruence. However, recent analyses reported the persistence of cross-taxon congruence when environmental effect was statistically removed. Here we evaluate this hypothesis, analyzing the cross-taxon congruence between birds and mammals in the Brazilian Cerrado, and assess the environmental role on the spatial covariation in diversity patterns.

**Results:**

We found a positive association between avian and mammal richness and a positive latitudinal trend for both groups in the Brazilian Cerrado. Regression analyses indicated an effect of latitude, PET, and mean temperature over both biological groups. In addition, we show that NDVI was only associated with avian diversity; while the annual relative humidity, was only correlated with mammal diversity. We determined the environmental effects on diversity in a path analysis that accounted for 73% and 76% of the spatial variation in avian and mammal richness. However, an association between avian and mammal diversity remains significant. Indeed, the importance of this link between bird and mammal diversity was also supported by a significant association between birds and mammal spatial autoregressive model residuals.

**Conclusion:**

Our study corroborates the main role of environmental conditions on diversity patterns, but suggests that other important mechanisms, which have not been properly evaluated, are involved in the observed cross-taxon congruence. The approaches introduced here indicate that the prevalence of a significant association among taxa, after considering the environmental determinant, could indicate both the need to incorporate additional processes (e.g. biogeographic and evolutionary history or trophic interactions) and/or the existence of a shared trend in detection biases among taxa and regions.

## Background

Spatial concordance in diversity of different taxa is a recurrent feature in nature [1-3]. The strength of this association, known as cross-taxon congruence, depends on the studied taxonomic groups and the scale of analysis [4]. This is because the spatial association in diversity patterns of related taxa is typically larger than that observed between weakly related taxa [5] and is more common over large geographic scales [6]. This scale dependence in the strength of spatial taxonomic covariation would be associated with changes in the factors determining species richness at different scales [7-9]. A finer spatial scale would increase the role of biological interactions like competition [10], which may lessen the spatial congruence between different groups. Several hypotheses have been proposed to explain the cross correlation between major taxa diversity. These can be grouped into those that focus on the diversity effect of one taxon on another taxon's diversity; those that propose that the diversity patterns of different taxa are determined by shared biogeographic history; and those that propose a common response to local environmental conditions. The former group includes the effect of trophic interactions on predator or prey diversity, to which particular attention has been devoted in the ecological literature [11-14]. However, empirical patterns of association between prey and predator diversity have shown inconsistent results [15-18]. Other mechanisms relate the diversity of one group to the resource availability, habitat and refuges that they provide for other groups, directly affecting their diversity [11,18,19]. Another explanation for cross-taxon congruence is based on their similar biogeographic history [20]. Allopatric speciation is typically related to a geographic structure that could simultaneously affect different taxa [21]. Geographic connection of areas previously isolated [22] or the long term variation in climatic conditions, produce large scale movements of several taxa which could generate congruent spatial distribution patterns among them [23,24].

A final set of explanations for spatial covariation in diversity patterns is rooted in their common response to environmental variability [3,25,26]. Climatic conditions have been broadly proposed as a major determinant of diversity patterns through their control over the availability of water and energy [1]. In this sense, species-energy theory [27] predicts that available energy determines viability of rare species and consequently community richness [14,28]. In addition, temperature could determine richness through its effects on vital rates [29,30]. In this framework, the observed cross-taxon congruence in diversity patterns would be a by-product of the similar response to spatial climatic variability. The large amount of evidence regarding the role of the environment as a determinant of geographic variations in diversity [17,25,31,32], lend support to common response to environmental conditions as a main explanation for cross-taxon congruence. A global study on the species richness determinants, showed that in 83 out of 85 studies, more than 60% of the spatial variability in species richness was explained by variables related with energy, water or their interaction [31]. However, the complex interaction among environmental variables constrains our ability to disentangle the determinants of variation in diversity within and among taxa [33,34]. In this article we focus on the evaluation of this mechanism as the main determinant of cross-taxon congruence, accounting for the complex nature of interaction among environmental variables which affect diversity.

The Brazilian Cerrado is the largest savannah of South America and has been recognized as one of the 25 biodiversity hotspots of the world [35]. A considerable number of studies have been conducted in this ecosystem analyzing its biogeographic patterns [36-38] and conservation status [39,40]. In the particular case of birds and mammals, a recent study showed that diversity of both groups is spatially correlated, showing a common latitudinal trend [41]. The results of this study also provide evidence for an important role of actual evapotranspiration as a common predictor of avian and mammal diversity patterns.

In this article, path analysis has been used to move forwards in the identification of the effect of environmental variables on avian and mammal richness, accounting for the potential existence of complex associations among variables. Once we had identified the independent effect of environment on taxon richness, we explored if environment could explain common spatial variations between taxa, as was proposed, or if this covariation should be accounted by additional factors.

## Methods

### Database

**Figure 1:**
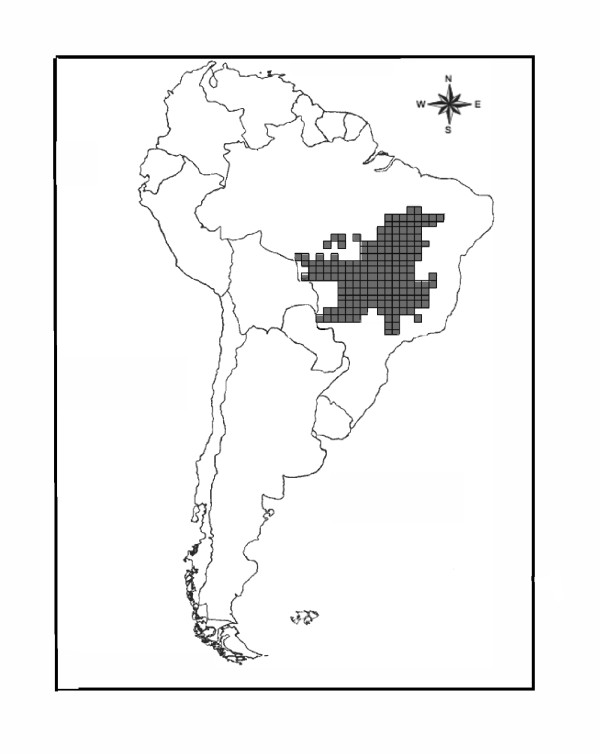
**Map of the Brazilian Cerrado with the used grid**. Map of the 181 grid cells (1° × 1°) over Brazilian Cerrado region.

The Cerrado (Brazilian Savanna), occupies an extension of approximately 2.000.000 km^2^, and represents the second largest biome of Brazil (Figure 1). This is the richest and most threatened savannah of the world [37]. Following previous studies the whole region was divided in 181 grid cells of one degree of spatial resolution [40,42,43]. For each grid cell we worked with the total species richness of birds and mammals. These richness values were estimated from the extent of occurrence of each species [44,45]. It was stated that spatial patterns of richness at the Cerrado could be affected by biases in data records associated with human occupancies. However, concerns for data quality are important for amphibians [46,47] but not so for the birds and mammals species used in this study [41,48]. In addition, the following environmental variables that could affect species richness were considered: mean temperature (Tmean), potential evapotranspiration (PET), normalized difference vegetation index (NDVI), annual relative humidity (ARH), and actual evapotranspiration (AET) [42]. The first three variables are typically used as alternative proxies of available energy in the environment. They are intended to represent the major hypotheses in the context of the species-energy theory [31]. Tmean and PET may be interpreted as a measure of crude ambient energy, although Tmean basically represents heat, and PET depends on a degree of relative humidity [1]. NDVI is a measure of productivity, and so it is a surrogate of the amount of energy that actually enters the food chains. The fourth variable is closely associated with water availability, and the last one represents the interaction between water and energy [43]. In addition, we also considered latitude-centre point of each grid cell-as a potential determinant of diversity, which encapsulates the variation in several environmental variables [34].

### Analysis

Our principal aim was to achieve a plausible causal structure connecting latitude, environment, birds and mammal richness and the correlation between them. We started by exploring the association between pairs of key variables, such as the association between avian and mammal diversity and the latitudinal trends of these taxa. The role of environmental variables was first analyzed with Ordinary Least Square (OLS) regression and Simultaneous Autoregressive (SAR) models. This analysis identified a set of variables that could be causal determinants of diversity patterns. We used those models as a starting point for the analysis of the association between birds and mammals diversity after accounting for environmental effects. We evaluated the importance of this mammals-birds link with two complementary approaches, whole covariance matrix path analysis [49] and d-separation [50].

The association between avian and mammal richness and latitudinal trends in diversity were explored by second order polynomic regressions [51]. Consideration of the second order term allows the detection of a wide range of non-linear relationships including U-shaped, unimodal, and monotonic trends [52]. We performed multiple regression analyses, also including quadratic terms for each independent variable, selecting models from ranked Akaike Information Criterion (AIC) values [53]. It has been highlighted that the result of macroecological analysis could be sensible to spatial autocorrelation in data [50,54]. For that reason we contrasted the results of the OLS regression with those observed in a SAR analysis [55].

Multiple regressions are limited in the range of causal structure that they can represent, and could even suggest a wrong model when a complex causality is involved [50]. Starting with the causal structure suggested by the previous regressions, we tested for alternative causal models using path analysis. This analysis proves the congruence of the whole causal model with observations and the significance of all proposed paths [50]. The whole model was evaluated with a maximum likelihood approach, using chi-square distribution (*χ*^2^) to statistically evaluate the congruence between observed and expected covariance with the proposed model. A significant result implies a lack of congruence between model and observations.

The potential existence of a causal link between mammal and avian diversity patterns is particularly important in the context of this study. If this link remains significant, in spite of having accounted for the existence of common determinants of diversity--environmental variables, available energy, and latitude--this implies that there is some other mechanism(s) determining the observed association. On the other hand, if the Structural Equation Model (SEM) is not improved for the inclusion of this link and/or the link is not significant this means that co-variation between higher taxa diversity could be fully accounted by geographic trends in environmental variables. We further evaluated the relevance of this link after considered spatial autocorrelation using d-separation [50]. For that, we explored the association between the residuals of SAR analysis for mammals and birds.

## Results

**Figure 2:**
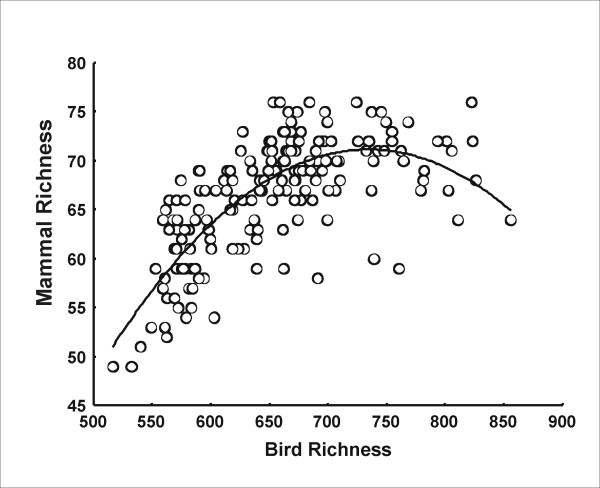
**Cross-taxon congruence of avian and mammal richness within the Cerrado region**. Relationships between avian and mammal richness in the Brazilian Cerrado. Line indicate the polynomic regression fit between groups (r^2 ^= 0.574, P < 0.05, N = 181).

**Figure 3:**
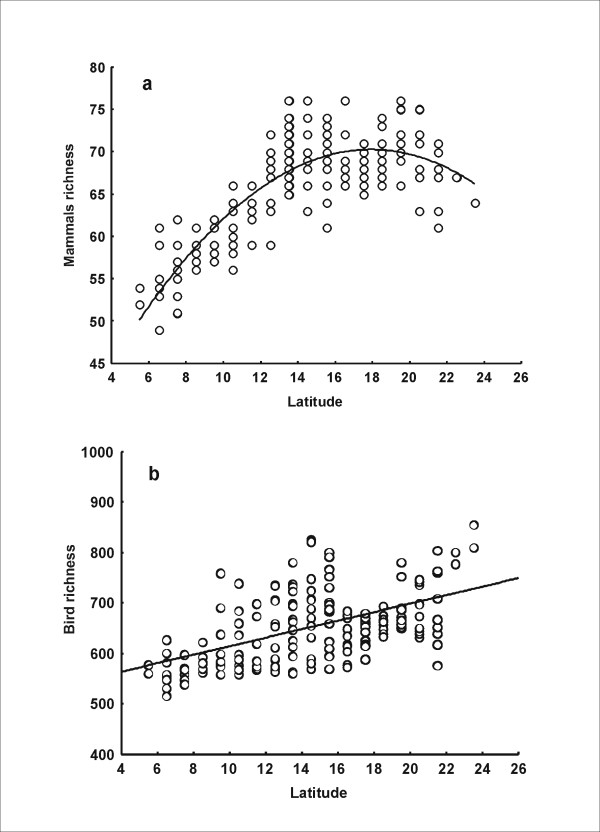
**Correlation of mammal and avian richness with Latitude in the Brazilian Cerrado**. Latitudinal trend of mammal (a) and birds (b) richness, within the Cerrado (Brazil).Lines indicates the regression fit of avian (r^2 ^= 0.292, P < 0.05, N = 181) and mammal diversity (r^2 ^= 0.689, P < 0.05, N = 181) with latitude.

Species richness of birds and mammals was positively associated in the Cerrado biome (r^2 ^= 0.574, P < 0.05, N = 181), as had been previously noted in a study on the spatial patterns of vertebrate diversity in this region [41]. The relationship between taxon richness was mainly positive and non-linear, but tended to be independent at high values of richness (Figure 2). Species richness of birds (r^2 ^= 0.292, P < 0.05, N = 181) and mammals (r^2 ^= 0.689, P < 0.05, N = 181) showed a positive latitudinal trend in the Brazilian Cerrado region. This result is consistent with diversity trends previously reported in this biome for amphibians [47] and birds [43]. The functional form of the latitudinal trend was different for birds and mammals. Mammal richness presented a positive but non-linear monotonic pattern (Figure 3a), while avian richness showed a positive linear relationship with latitude (Figure 3b).

**Figure 4:**
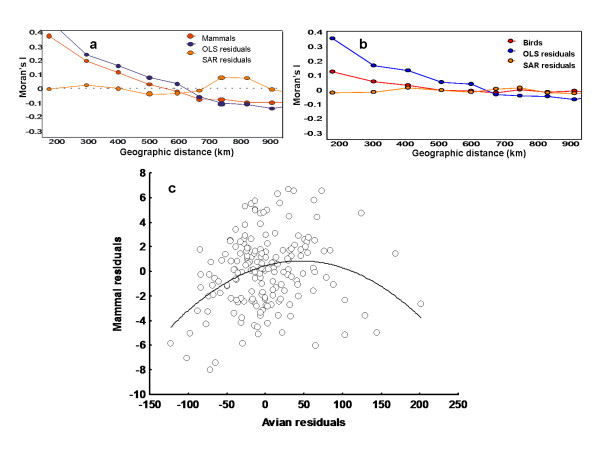
**Cross-taxon congruence in birds and mammals after accounting for the effect of the environment and spatial structure**. Spatial correlograms of mammals (a) and birds (b) richness red lines-richness data; blue lines-OLSs residuals and orange lines-SARs residuals; (c) association between residuals of SARs analyses for mammals and birds (r^2 ^= 0.109, P < 0.05, N = 181).

OLS regression indicates an effect of latitude and its quadratic term, PET, mean temperature and its quadratic value, over both taxa. In addition, NDVI was only associated with avian diversity; while the annual relative humidity, was only related with mammal diversity (Table 1). It should be highlighted that the potential role of AET was evaluated in all multiple regression models, but its inclusion always implied a significant decrease in model performance--e.g. more than two units of difference in AIC value between models. The results of SAR models were largely congruent with previous OLS results (see Table1). However, OLS showed a significant spatial structure on residuals which was removed in the SAR when the spatial components were considered (Figure 4a y 4b).

**Table 1:** Exploratory analyses of the effects of environment on diversity of birds and mammals.

	**OLS coeff**.	**SAR coeff**.	Std. Coeff	Std. Error	P-value
**Birds**					
Latitude	10.555	10.741	0.681	1.448	<0.001
Latitude 2	-0.733	-0.736	-0.221	0.228	0.002
PET	-1.998	-2.006	-0.39	0.532	<0.001
Tmean	28.322	28.079	0.683	5.407	<0.001
Tmean 2	7.774	7.666	0.504	1.169	<0.001
NDVI	16.275	16.062	0.17	5.13	<0.001

**Mammals**					
Latitude	1.043	1.058	0.783	0.088	<0.001
Latitude 2	-0.123	-0.12	-0.419	0.014	<0.001
PET	-0.093	-0.088	-0.2	0.032	0.006
Tmean	1.331	1.27	0.36	0.32	<0.001
Tmean 2	0.133	0.132	0.101	0.07	<0.001
ARH	0.271	0.272	0.197	0.052	<0.001

Birds R^2 ^(SAR): 0.515 Mammals R^2 ^(SAR): 0.745					

Spatial autoregressive analyses exploring the role of environmental conditions on avian richness and mammal richness. Lat = latitude, Lat^2 ^= quadratic term of latitude, PET = potential evapotranspiration, Tmean = mean temperature, Tmean^2 ^= quadratic term of mean temperature, NDVI = normalized difference vegetation index and ARH = annual relative humidity.

**Figure 5:**
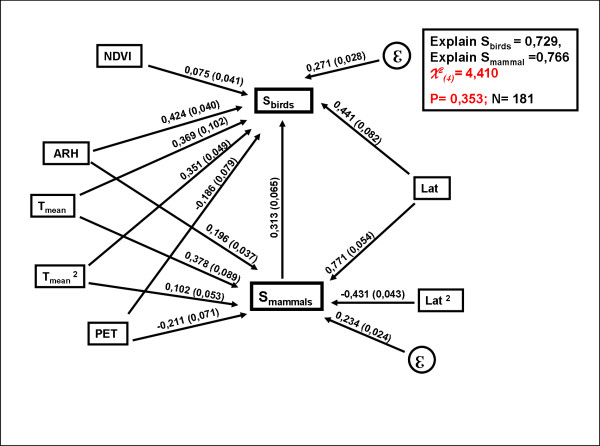
**Causal structure connecting environmental conditions, latitude, and diversity of mammals and birds**. Structural equation model connecting environmental conditions, latitude, and diversity of mammals and birds.The overall model was non significant indicating good congruence between the proposed causal structure and observations. Path model with the environmental determinants of bird (NDVI, HRA, PET, Tmean, Lat) and mammal richness (HRA, PET, Tmean, Lat and Lat^2^) and the remaining direct link between biological groups. Above the arrows it is shows the path coefficients, with its standard error in parentheses.

We identified a structural model congruent with observations, accounting for the interactions of the environmental variables and their roles as determinants of avian and mammal richness. The whole model was not significant (p < 0.353), indicating a good adjustment with the observations. This model explained 73% and 76% of geographical variability of birds and mammals (Figure 5). This final model retains an explicit connection between the taxa, once abiotic variability is controlled for. It should be highlighted that all alternative models analyzed were incongruent with observations if this path was not considered (Figure 5). Finally, it is important to state that significant paths between environmental variables were suggested by some models. This suggests the potential existence of some complex interaction among environmental variables. However, all these causal models were significantly different from the data, strongly supporting the final model.

The d-separation analysis of the mammals -birds' link was congruent with path analysis results. Residuals of the SAR models for each taxon were significantly associated (F_2,178 _= 10.86, P < 0.001). However, this analysis highlights that the nature of this relationship could be humped, being negative for the larger values of bird richness (Figure 4c).

## Discussion

Environmental variables have been broadly recognized as main determinants of geographic diversity patterns. Previous studies, using alternative methodologies, identified: temperature, elevation range, and actual or potential evapotranspiration as determinants of avian and mammal richness [31,32]. Our analyses corroborate a main influence of environmental conditions and latitude over these groups diversity [41]. Explanations for cross-taxon congruence based on common response to environmental conditions propose that the spatial concordance in diversity patterns is a consequence of the congruent response to environmental determinants [3,56,57]. Therefore, once the effect of environment on taxa diversity has been accounted for, such covariation should not persist [50]. The prevalence of a significant association among taxa, after considering environmental determinants, could indicate both the need to incorporate additional ecological mechanisms and the existence of a shared trend in the sampling bias among taxa and regions.

Spatial association in the data record is a typical source of biases in geographic databases [58-61]. Spatial biases are primarily generated by higher sampling effort along riversides and roads, as well as around populated centres [59,62,63]. At least for anurans, this has been shown to be a relevant problem in the Cerrado region [47]. These biases are expected to be lower in birds and mammals in this region, given that their accumulation curves were early saturated (Diniz-Filho, pers. *comm*. 2010). However, the existence of large areas poorly sampled and others very well recorded, could produce artificial shared trends in diversity patterns and hence cross-taxon congruence. The detection of a significant association between taxa, not related to environmental conditions, suggests that spatial biases in sampling could be determining the observed patterns. It should be highlighted that this explanation for cross-taxon congruence is not usually considered, despite spatial biases in databases being a common feature.

Recent studies have also shown the persistence of cross-taxon congruence when the environmental effects on taxa diversity have been controlled [19,64-67]. This evidence suggested the existence of a functional links between plant and vertebrate richness and among vertebrate groups. In this sense, a recent analysis of cross-taxon congruence at a global scale reported the presence of a strong link between avian and mammal diversity [65]. However, links between these groups' diversity and diversity of vascular plants and other vertebrate taxa were weak [65]. All these studies support two basic ecological hypotheses, the diversity-trophic structure hypothesis [14] and the vegetation structure hypothesis [68]. These hypotheses attempt to explain the diversity of certain groups as an effect of other group diversity. In the particular case of birds and mammals at the Brazilian Cerrado, habitat supply from one taxon to the other does not appear as plausible explanation. However, indirect effects on landscape attributes as generation of habitat heterogeneity or particular habitat condition could be involved in the observed pattern. In addition, trophic interaction could be playing a role in this case. In spite of representing a small number of the total species in both groups, top down effects from raptors and predatory mammals have the potential to determine prey diversity. In this sense, we are not considering a priority effect of one group's diversity on the other but the persistence of their association after accounting for the effect of the shared environment. Although the effect of available energy was considered in the analysis, bottom up processes could be implicated because the availability of some resources may be poorly represented by gross measures of productivity [69]. Particularly, the response of some guilds composed of birds and mammals to variations in resources may originate spatial covariation between taxa [70,71].

Common biogeographic history could cause the persistence of cross-taxon congruence. The role of biogeographic history as a determinant of current spatial patterns of diversity is increasingly recognized [21-24,72,73]. In the particular case of South America, evidence has been compiled about the effects of past climate fluctuations in the contraction and expansions of tropical and temperate habitats [74], and the consequences of these changes on regional taxa diversity [20,38,75,76]. Further, it has been postulated that the Quaternary climatic and vegetational changes have been an important driver of the process of speciation in the forests and savannah in South America [37]. The evidence for the expansion and contraction of the savannas and dry forests in the Cerrado and Caatinga [36,38], suggest a potential influence of biogeographic history on the cross-taxon congruence herein reported.

The set of mechanisms discussed above represents a plausible explanation for cross-taxon congruence between birds and mammals. However, it remains to be considered why these groups present a common response to these mechanisms, determining cross-taxon congruencies that are larger that those observed with any other group of vertebrates [65]. The most important attribute shared by birds and mammals is endothermy [77]. Endothermy implies a very expensive lifestyle, consuming energy at higher rates than reptiles [78]. In addition, endothermy is related to different ecological attributes than is vertebrate ecothermy [77]. This is the case for density [30], reproductive behavior [79], efficiency in translation of available resources to reproduction [77], the strength of predator-prey size differences [80], a hierarchy in which endotherms prey on ecthoterms [81], maximum body size and trophic position [82], molecular evolution [83] and current extinction rates [84]. The distinctive ecology of endotherms could be involved in the similar response of birds and mammals to ecological and evolutionary processes. Indeed, high cross-taxon congruence also has been observed among ectothermic vertebrate, reptiles and amphibians in terrestrial ecosystems [24,41,65].

Although that for most of the data considered the association between mammal and bird richness was positive, observed richness in both groups tended to be independent at higher richness (Figure 2), or, even became negative when residuals of SAR regressions were considered (Figure 4). This puts the attention on those mechanisms proposed to explain observed incongruence in both groups' diversity in some regions of the Cerrado. In this sense, previous studies have suggested that differences in birds' and mammals' diversity patterns can be based in the more restricted distribution ranges of mammals in southern and western zones of the biome [41] or could be produced as a consequence of difference in ecological requirements related to habitat use [43]. In summary, several hypotheses have been proposed for the incongruence in diversity of birds and mammals at some points of the Cerrado. However, the potential existence of negative trends among groups diversity have been poorly considered as a general phenomenon. Since cross-taxon congruence is usually invoked as a theory that supports the implementation of reserves based on groups that act as surrogates for the diversity of other taxa [35], more attention should be put on the determinants of these negative trends (see also [67]).

In the case of common environmental determinants the most frequent methodological approach has been multiple regression models [31,34,85]. Despite the fact that this approach represents a breakthrough in the analysis of associations between environment and diversity at broad spatial scales, it has limited potential to identify complex causal structures [86]. The use of structural equation models allows the detection of these intricate structures [49,50], which combined with statistics that account for spatial autocorrelation, could significantly improve our understanding of the mechanisms determining diversity patterns. In the present study, these analyses allowed us to identified the role of environmental variables as determinants of observed richness, as well as to discard a common environmental effect as the only determinant of avian and mammal covariation.

## Conclusions

The role of environment as a main determinant of diversity within particular taxa has been identified as the primary cause of spatial congruence between different groups diversity. Contradicting previous expectations, the analysis of cross-taxon congruence in different systems suggests that a common response to environmental variables cannot completely account for the observed associations among taxa diversity [64,65]. The identification of those mechanisms that, in addition to environment, determine cross-taxon congruence has important implications for the understanding of ecological patterns, and ongoing process as global change, species extinction and conservation.

## Authors' contributions

Both authors contributed to the conception and design of the study. Also, CT and MA were involved in the statistical data analyses and interpretation, drafting and revising the article. All authors approve the article's publication.

## Authors' information

CT is currently in the Institute of Ecology and Environmental Sciences, Universidad de la República, Montevideo, Uruguay. MA is currently in the Department of Ecology and Evolution, Universidad de la República, Montevideo, Uruguay and is an associated researcher in the Center for Advanced studies in Ecology and Biodiversity, Pontificia Universidad Católica de Chile.
